# The *C. elegans *EMAP-like protein, ELP-1 is required for touch sensation and associates with microtubules and adhesion complexes

**DOI:** 10.1186/1471-213X-8-110

**Published:** 2008-11-17

**Authors:** Jennifer L Hueston, Gina Purinton Herren, Juan G Cueva, Matthew Buechner, Erik A Lundquist, Miriam B Goodman, Kathy A Suprenant

**Affiliations:** 1 Department of Molecular Biosciences, University of Kansas, Lawrence, KS, USA; 2 Molecular and Cellular Physiology, Stanford University School of Medicine, Stanford, CA, USA

## Abstract

**Background:**

The founding member of the EMAP-like protein family is the Echinoderm Microtubule-Associated Protein (EMAP), so-named for its abundance in sea urchin, starfish, and sand dollar eggs. The EMAP-like protein family has five members in mammals (EML1 through EML5) and only one in both *Drosophila *(ELP-1) and *C. elegans *(ELP-1). Biochemical studies of sea urchin EMAP and vertebrate EMLs implicate these proteins in the regulation of microtubule stability. So far, however, the physiological function of this protein family remains unknown.

**Results:**

We examined the expression pattern of *C. elegans *ELP-1 by means of transgenic gene expression in living embryos and adults, and by immunolocalization with an ELP-1-specific antibody in fixed tissues. In embryos, ELP-1 is expressed in the hypodermis. In larvae and adults, ELP-1 is expressed in the body wall, spermatheca and vulval muscles, intestine, and hypodermal seam cells. In muscle, ELP-1 is associated with adhesion complexes near the cell surface and is bound to a criss-crossing network of microtubules in the cytoplasm. ELP-1 is also expressed in a subset of mechanoreceptor neurons, including the ray neurons in the male tail, microtubule-rich touch receptor neurons, and the six ciliated IL1 neurons. This restricted localization in the nervous system implies that ELP-1 plays a role in mechanotransmission. Consistent with this idea, decreasing ELP-1 expression decreases sensitivity to gentle touch applied to the body wall.

**Conclusion:**

These data imply that ELP-1 may play an important role during the transmission of forces and signals between the body surface and both muscle cells and touch receptor neurons.

## Background

Microtubule networks are necessary for a variety of essential processes including cell polarity, migration, division and mechanotransduction [1-3]. Microtubule formation and function is regulated by a variety of proteins that mediate the structural and regulatory interactions between these microtubules and their cargo [4,5], and catalyze their assembly and disassembly [6]. In this report we examine the tissue-specific expression and the subcellular location of ELP-1, an EMAP-like protein from *C. elegans *and demonstrate that ELP-1 contributes to touch-sensitivity in a subset of mechanoreceptor neurons.

The founding member of the EMAP-like protein family is the 75 kDa Echinoderm Microtubule-Associated Protein (EMAP), so-named for its abundance in sea urchin, starfish, and sand dollar eggs [7]. Genes that code for EMAP-like proteins are conserved in the genomes of nematodes, chordates, insects, echinoderms, and platyhelminthes and several EMAP-like proteins have been shown to bind to microtubules *in vitro *and *in vivo *[8-13].

The *in vivo *function of EMAP and EMAP-like proteins is unknown. However there are indications that loss or alteration of EMAP function may lead to human disease. Human EML2 RNA is abundant in cancer cell lines including chronic myelogenous leukemia (K-562), lymphoblastic leukemia (MOLT-4), colorectal adenocarcinoma (SW480), and lung carcinoma (A549) [11]. Furthermore, in certain patients with T-cell acute lymphoblastic leukemia (TALL), the gene encoding the nonreceptor protein kinase (c-ABL1) is fused to the EML1 gene on chromosome 14, which causes expression of an EML1-ABL1 fusion protein, that functions as a dysregulated tyrosine kinase [14]. The EML1-ABL1 fusion protein constitutively activates the ERK, Stat5, and Src signalling pathways. The N-terminal coiled-coil domain of EML1 is required for kinase activation, which suggests that oligomerization of EML1 is required for the function of EML1-ABL1 fusion proteins.

Kinase oncogenes are not restricted to fusions with EML1. EML4 fusions with the anaplastic lymphoma kinase (ALK) occur in a subset of non-small cell lung cancers and adenocarcinomas of the lungs [15,16]. The amino portion of EML4 (residues 1–496) is fused to residues 1058–1620 of the intracellular domain of the ALK tyrosine kinase. The basic amino terminal domain of EML4 is critical to the catalytic activity of the ALK fusion [16]. These results imply that EML-kinase fusions and rearrangements may underlie other acquired solid tumours and blood related cancers.

The conservation of the EMAP-like protein family amongst metazoans and the direct correlation between EML translocations and cancer indicates that this novel protein family may perform an important function in cells and tissues. To begin to understand the function of EMAP and EMAP-like proteins we undertook a molecular and cytological analysis of the *elp-1 *gene encoded by the ORF *F38A6.2 *in *Caenorhabditis elegans*. We determined whether ELP-1 bound to microtubules *in vitro *and *in vivo*. Furthermore, we took advantage of the transparency of the worm to examine the expression pattern of an ELP-1::GFP fusion protein in embryos and in adults. Our results indicate that ELP-1 is expressed in cells that make productive interactions with the extracellular matrix, including but not limited to the hypodermis, body wall muscles, male-specific sex muscles, and the microtubule-rich touch receptor neurons. In addition, our behavioural studies show that wild-type levels of ELP-1 are needed for touch sensation in the worm.

## Results

### *elp-1 *encodes the sole *C. elegans *member of the EMAP-like protein family

**Figure 1:**
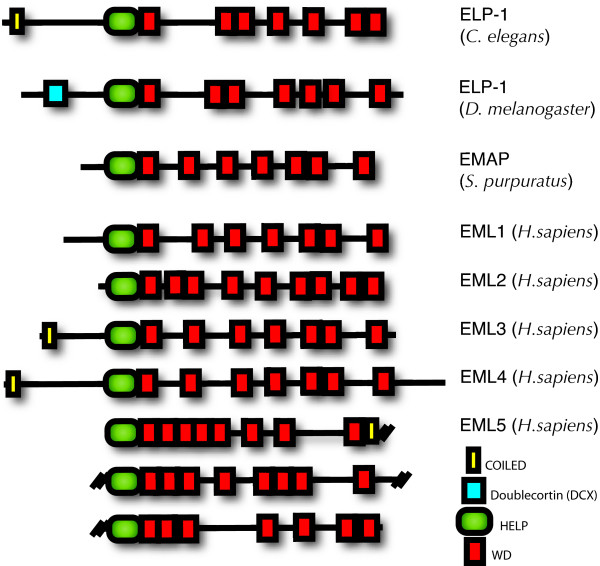
**ELP-1 domain structure.** All members of the EMAP-like protein family are constructed with a Hydrophobic ELP (HELP) motif (PF03451) preceding a WD domain (PF00400). A COILED domain (PF05710) is predicted in *C. elegans *ELP-1 as well as in three of the five human EMLs. An additional doublecortin (DCX) domain (PF 03607) is predicted in fruit fly ELP-1. Accession numbers for the domains/motifs (parentheses) are provided from the Protein Family (PFAM) database [57]. The accession numbers for the human EMLs are as follows: EML1 [GenBank: NM_001008707], EML2 [GenBank: NM_012155], EML3 [GenBank: NM_153265], EML4 [GenBank: NM_019063], and EML5 [GenBank: NM_183387].

All of the EMAP-like proteins identified to date, including vertebrate EMLs and invertebrate ELPs, share a common domain organization with a short, 60–70 amino acid, hydrophobic EMAP-like protein (HELP) motif preceded by a series of WD repeat domains (Figure 1). Although the function of the HELP motif is unknown, it is unique to this gene superfamily. There is evidence in the human genome for 6–8 EML genes, however at this time only EMLs 1–5 have a confirmed gene product. The apparent or predicted molecular mass of most EMAP-like proteins ranges from ~70 to ~120 kDa (Figure 1). Human EML5 is the exception with a predicted Mr of 220 kDa and three repeats of the HELP and WD domains.

**Figure 2:**
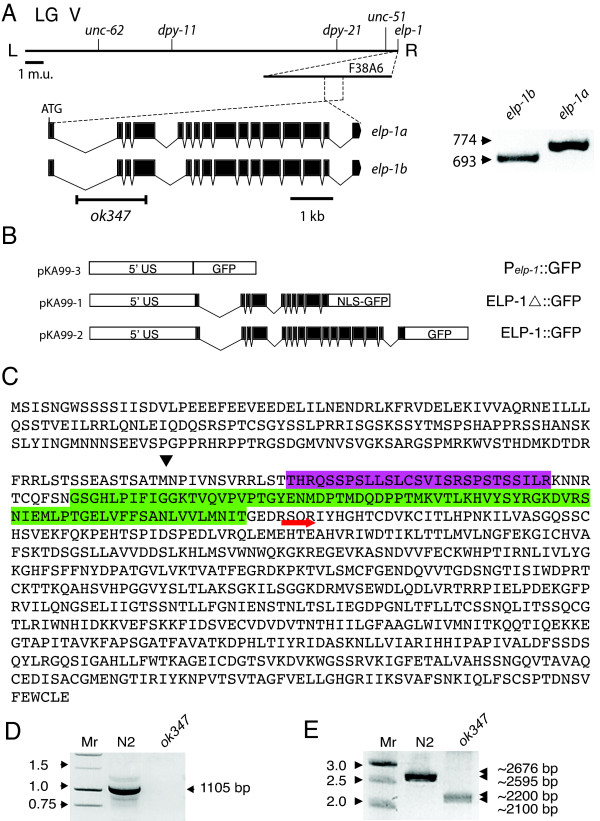
**Genetic and molecular characterization of *elp-1*.** (A) The physical map of *elp-1* is drawn in the reverse orientation.  Two alternatively spliced transcripts, *elp-1a* and *elp-1b*, are shown with exons as solid boxes and introns as lines.  We confirmed the identity of the PCR products by a diagnostic restriction digest with *EcoR1* and *BglII* (Panel A, right). (B) Genetic structures of the *elp-1::gfp* expression constructs pKA99-1, pKA99-2 and pKA99-3. Exons are indicated by black boxes. The proteins encoded by these constructs are indicated as P_elp__-1_::GFP, ELP- 1Δ::NLS::GFP, and ELP-1::GFP, respectively in the Text. (C) Amino acid sequence of ELP-1a showing a potential alternative start site for *elp-1(ok347)* (black arrow),   exon 5 aa sequence (magenta),  HELP motif (green) and the beginning of the WD domain (red arrow). (D) PCR reaction with a forward primer in the deletion site and a reverse primer in exon 11.  There is no detectable wild-type copy of the *elp-1* gene in the deletion strain as evidenced by the absence of the 1105 bp band. (E)  The *elp-1(ok347)* strain retains two mRNAs of approximately 2200 and 2100 bp.

The *C. elegans *genome contains a single EMAP-like gene, *elp-1*, that maps to the right of *unc-51 *on the extreme right arm of linkage group (LG) V (Figure 2). Genefinder™ analysis predicts that the *elp-1 *gene consists of 16 exons that encode an 891-amino acid polypeptide of 98 kDa. We used reverse transcription coupled to the polymerase chain reaction (RT-PCR) to show that there are at least two alternatively spliced variants: a full-length cDNA transcript composed of exons 1 through 16 (*elp-1a*); and an alternatively spliced transcript composed of 15 exons (*elp-1b*). The *elp-1b *transcript lacks exon 5, an 81 bp segment that encodes for a 27-amino acid sequence that is more than 46% serine and threonine residues (Figure 2C). The *elp-1b *transcript is identical to the cDNA EST yk209e10 that is predicted to be translated into an 864-amino acid polypeptide of ~95 kDa [17].

Exon 5 is conserved in nematodes but not detected in other EMAP-like proteins. Twenty-four of the twenty-seven predicted amino acids (88%) are identical in *Caenorhabditis briggsae *and *C. elegans*. All three substitutions are conservative (V15I, I16V, and S19N; numbers refer to the 27 amino acids coded by this exon). This domain shows a slightly higher level of sequence conservation when compared to the HELP domain protein sequence (81% identical) and the full-length protein sequence (80% identical) of *C. elegans *and *C. briggsae*. Although the function of this 27 amino acid region, rich in potential phosphorylation sites, is unknown, these observations indicate that exon 5 may be important for a nematode-specific function.

To learn more about the function of *elp-1*, we obtained a deletion allele, *ok347*, from the *C. elegans *Knockout Consortium. The *ok347 *allele deletes 1301 nucleotides, spanning intron 1 through intron 4 (Figure 2A, 2D). Starting from the ATG of the ELP-1a open reading frame, *ok347 *is missing base pairs 421 to 1721. This genome deletion does not remove ELP-1 function however, since *elp-1(ok347) *worms retain two nearly full-length transcripts (Figure 2E). These transcripts are predicted to encode two proteins that include both the HELP and WD repeat domains, a finding which implies that these proteins could retain partial function.

### Expression patterns of the *elp-1 *gene in embryos and adults

To learn how ELP-1 contributes to development, physiology and behaviour, we examined the expression pattern of the *elp-1 *gene. Three GFP reporter constructs were generated: an ELP-1 promoter construct [pKA99-3], a truncated ELP-1 with an NLS [pKA99-2]; and a full-length ELP-1 [pKA99-1] (Figure 2B). All three constructs were expressed in the same cells and tissues. For clarity, the protein expressed from the pKA99-1 construct will be noted as ELP- 1Δ::NLS::GFP and the full-length protein expressed from the pKA99-2 construct will be written as ELP-1::GFP.

**Figure 3:**
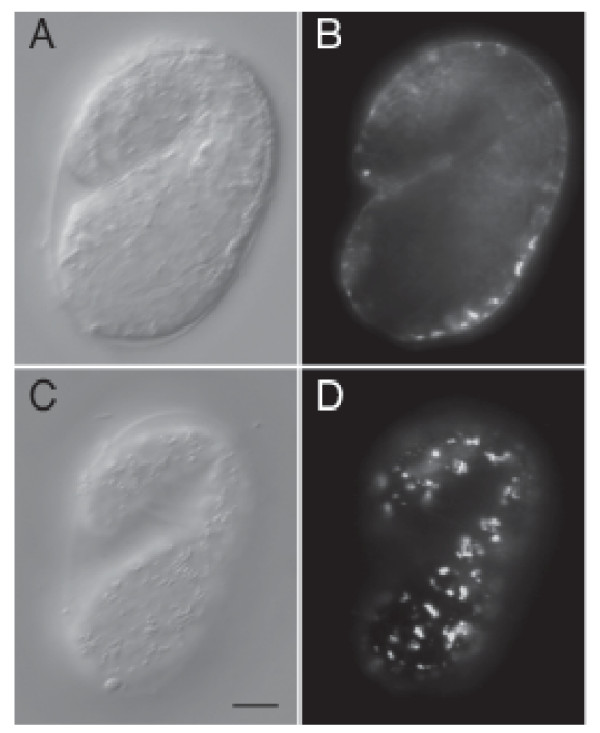
**ELP-1::GFP is expressed in hypodermal cells at the 1 1/2-fold stage of development.** Two focal planes within the same embryo are examined by differential interference contrast (DIC) (A, C) and fluorescent light microscopy (B, D). The bar represents 10 μm.

**Figure 4:**
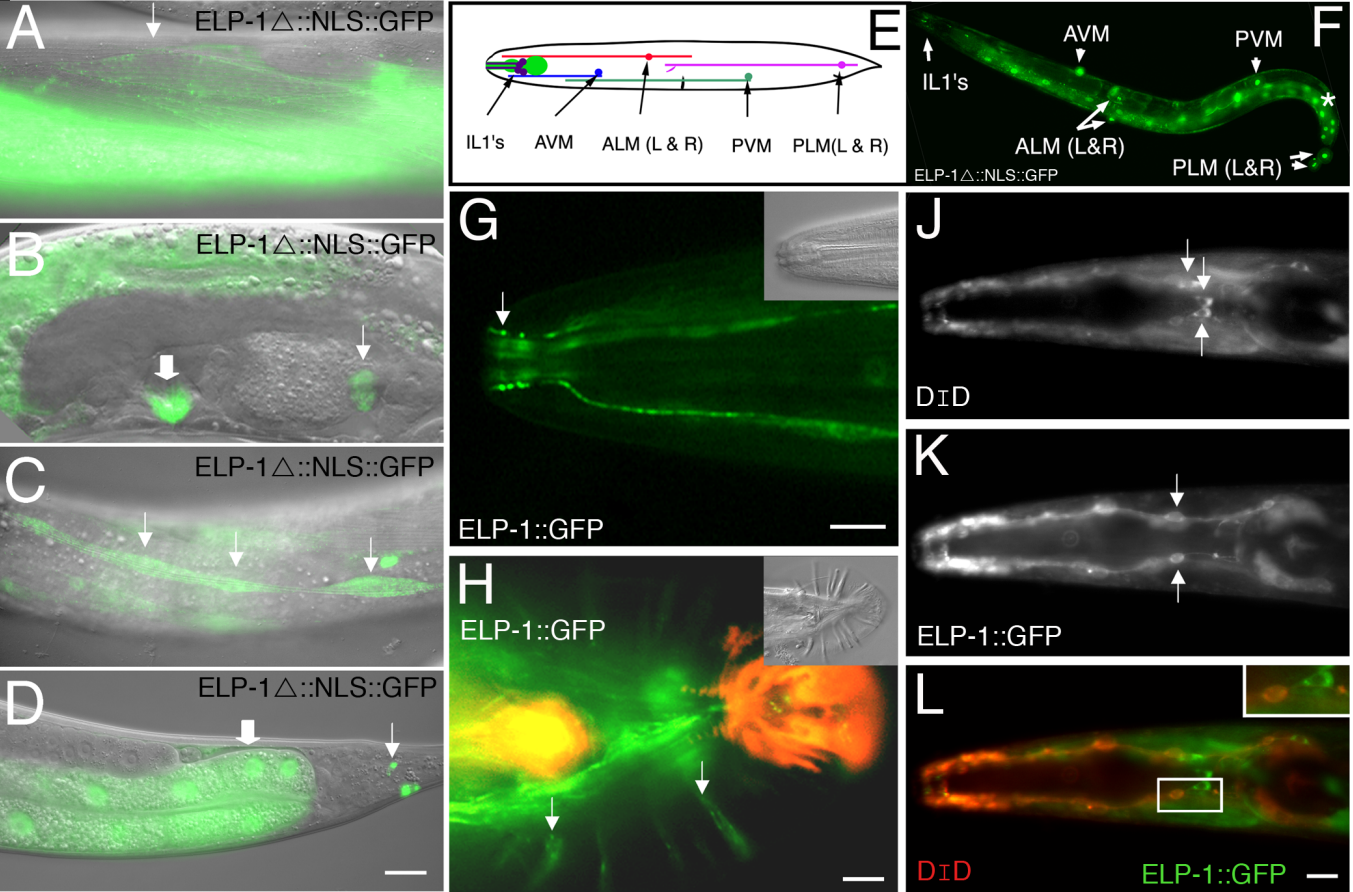
**Expression of ELP-1 in adult cells and tissues.** (A-D) DIC micrographs were overlaid with fluorescent images to show expression of ELP- 1Δ::NLS::GFP in body wall muscle (A, arrow), vulva muscle cells (B, thick arrow) and the filamentous spermathecal valve (B, thin arrow), hypodermal seam cells (C, thin arrows), intestines (D, thick arrow), and tail neurons (D, thin arrow). Panels E and F show ELP- 1Δ::NLS::GFP expression in all six touch receptor neurons (ALML & R; PLML & R; AVM & PVM).  Intestinal nuclei are visible in this plane of focus (asterisk).  In panel G, ELP-1::GFP protein is shown  in the ciliated endings of a group of head neurons that are identified in Panels J-L. In addition, ELP-1::GFP is prominently expressed in all nine mechanoreceptor ray neurons of the male tail (Panel H, arrows).  DIC images are shown as insets.  The yellow and orange fluorescence in the male tail is autofluorescence. In Panels J-L, the head neurons in Panel G are identified as the IL1 neurons.  DiD-filled IL2 cells (J, L) are located anterior to ELP-1::GFP expressing neurons (K, L).  Bars represent 10 μm.

In embryos, ELP-1::GFP expression first appeared during the comma stage. As the embryo matured into the 1-1/2 fold stage, the strongest expression was seen in the hypodermal cells, with only diffuse staining throughout the rest of the embryo (Figure 3). During larval development, expression of the fusion protein was progressively refined to muscle, neurons and epithelial cells. In the adult, ELP-1::GFP and ELP- 1Δ::NLS::GFP were expressed in body wall muscle, spermatheca, vulval muscle, seam cells, the intestine, touch receptor neurons (TRNs) and inner labial 1 (IL1) neurons of the head. Expression of ELP- 1Δ::NLS::GFP is shown in Figure 4A–D. The truncated ELP-1 construct with the NLS was used in order to identify the neuronal cell bodies.

### ELP-1 is expressed in a subset of mechanoreceptor neurons

As indicated above ELP-1::GFP and ELP-1Δ::NLS::GFP were expressed in cells that were identified as mechanoreceptor neurons (Figures 4E &4F). Although the NLS did not restrict expression to the nucleus, it was generally easier to identify neurons with this construct than with the full-length ELP-1::GFP construct. Specifically, ELP-1Δ::NLS::GFP was found in the six touch receptor neurons (ALML/R, AVM, PVM, and PLML/R) responsible for detecting light touch applied to the body surface (Figure 4E, F) [18]. These cells were identified by the position of their cell bodies and the long nerve cell processes that extend anteriorly or posteriorly over half of the body length. Touch receptor neurons (TRNs) are not ciliated or organized into sensilla, but extend neurites along the midline and lateral line of the worm with their dendritic receptors lying within 150 nm of the inner border of the cuticle [19].

ELP-1::GFP was also expressed in the dendrites and the ciliated endings of six neurons in the head (Figure 4G, J–L). Candidates for these six neurons included the six inner labial 1 neurons (IL1s), six inner labial 2 neurons (IL2s), and six outer labial neurons (2OLLs and 4 OLQs) [20]. To determine the identity of these neurons, the worms were labeled with the lipophilic tracer molecule DiD which gains access to the sensory cilia that project through an opening in the cuticle. In the presence of calcium acetate, DiD is exclusively taken up into the six IL2 neurons, although occasional dye-filling of the amphid neurons can be observed (personal communication, Elizabeth Ryder, Worcester Polytechnic Institute). The ciliated nerve endings of IL2 and IL1 both extend through the inner labial sensillum. However, only the IL2 nerve endings extend through a hole in the cuticle and are able to take up the dye. Dye-filling in the presence of calcium acetate allowed visualization of the IL2 neurons and thus allowed for a comparison to be made between the position of the IL2 neurons relative to the ELP-1::GFP expressing neurons (Figure 4J–L). DiD staining in the presence of calcium acetate showed that the IL2 neuronal cell bodies were located anterior to the ELP-1::GFP expressing neurons. This finding suggests that these head neurons are the mechanosensory IL1 neurons. We confirmed this identification by crossing ELP-1::GFP worms with *deg-1(u38) *mutations. The toxic DEG-1 protein caused the degeneration of the neurons which normally express the *deg-1 *gene: IL1, PVC, AVG and AVD [21]. In the *deg-1(u38) *background, we found fewer than three GFP- labelled neurons in the head (Table 1). These results confirm that the IL1 mechanosensory neurons express ELP-1::GFP.

**Table 1:** Quantification of IL1 neurons in a *deg-1 *mutant background.

Strain	Genotype	IL1 neurons/worm	Total number of worms
KA15	lkEx4[P_elp-1_::elp-1(Δ11–16)::nls::gfp; rol-6(su1006)]	5.4 + 0.7	30
KA37	deg-1(u38); lkEx4[P_elp-1_::elp-1(Δ11–16)::nls::gfp; rol-6(su1006)]	1.9 + 1.2	30

The number of IL1 neurons per worm was determined for the lkEx4[*P_elp-1_::elp-1(Δ11–16)::nls::gfp*, *rol-6(su1006)*] strain alone and for the lkEx4[*P_elp-1_::elp-1(Δ11–16)::nls::gfp*, *rol-6(su1006)*] strain in the *deg-1(u38) *dominant gain-of-function mutant background. Results are reported as number of IL1 neurons per worm +/- the standard deviation. p < 0.001 from a one-tailed *T*-test.

In addition to the mechanoreceptor neurons described above, ELP-1::GFP was associated with the nine pairs of rays of the male tale (Figure 4H). The male tale is a sensory apparatus used to find the hermaphrodite vulva during mating. The relatively diffuse and broad band of fluorescence appears to originate from the hypodermal cells (hyp7) and one or more of the neuronal cells of the ray (RnA, RnB).

### ELP-1 is associated with adhesion sites

**Figure 5:**
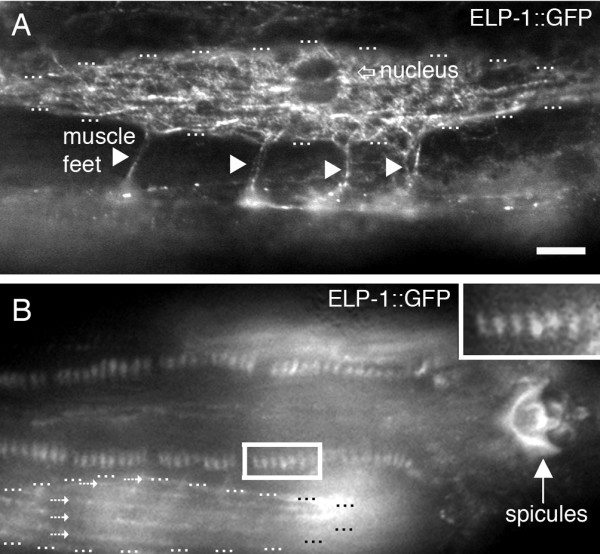
**ELP-1 is expressed in diverse muscle cellular junctions.** (A) A triplet of dots surrounds the fusiform-shaped body wall muscle cell. ELP-1::GFP is located throughout the muscle arms (arrowheads) that make junctions with the ventral nerve cord. The nucleus is shown in this plane of focus. (B) ELP-1::GFP is located at the adhesion sites of the male-specific sex muscles (boxed insert). The auto-fluorescent spicules of the male tail are visible. A body wall muscle cell is observed below the sex-muscle adhesion sites. The cell is outlined with triplet dots. Dotted arrows show a linear expression pattern in this body wall muscle cell. Bar represents 10 μm.

At the subcellular level, ELP-1::GFP was associated with adhesion sites in both males and hermaphrodites. In the hermaphrodite body wall muscle, ELP-1::GFP was associated with the muscle arms that terminate in neuromuscular junctions (Figure 5). The muscle arms are long cellular processes that extend to make contact with motor axons. The muscle cell shown in Figure 5A shows four muscle arms, which is consistent with the three to five muscle arms classically observed per body wall muscle cell [22,23]. The ELP-1::GFP fluorescence is found throughout the muscle arm.

Figure 5B shows a male with ELP-1::GFP associated with repeating focal attachment points in the sex-specific muscles of the worm. These muscles are located in the posterior end of the worm and function in various phases of male mating behaviour [24]. ELP-1 expression was prominent at the adhesion sites located at the sarcolemma. Unlike the dense body adhesion sites described below, these sites occur at the muscle ends in a plane perpendicular to the long axis of the sarcomere.

**Figure 6:**
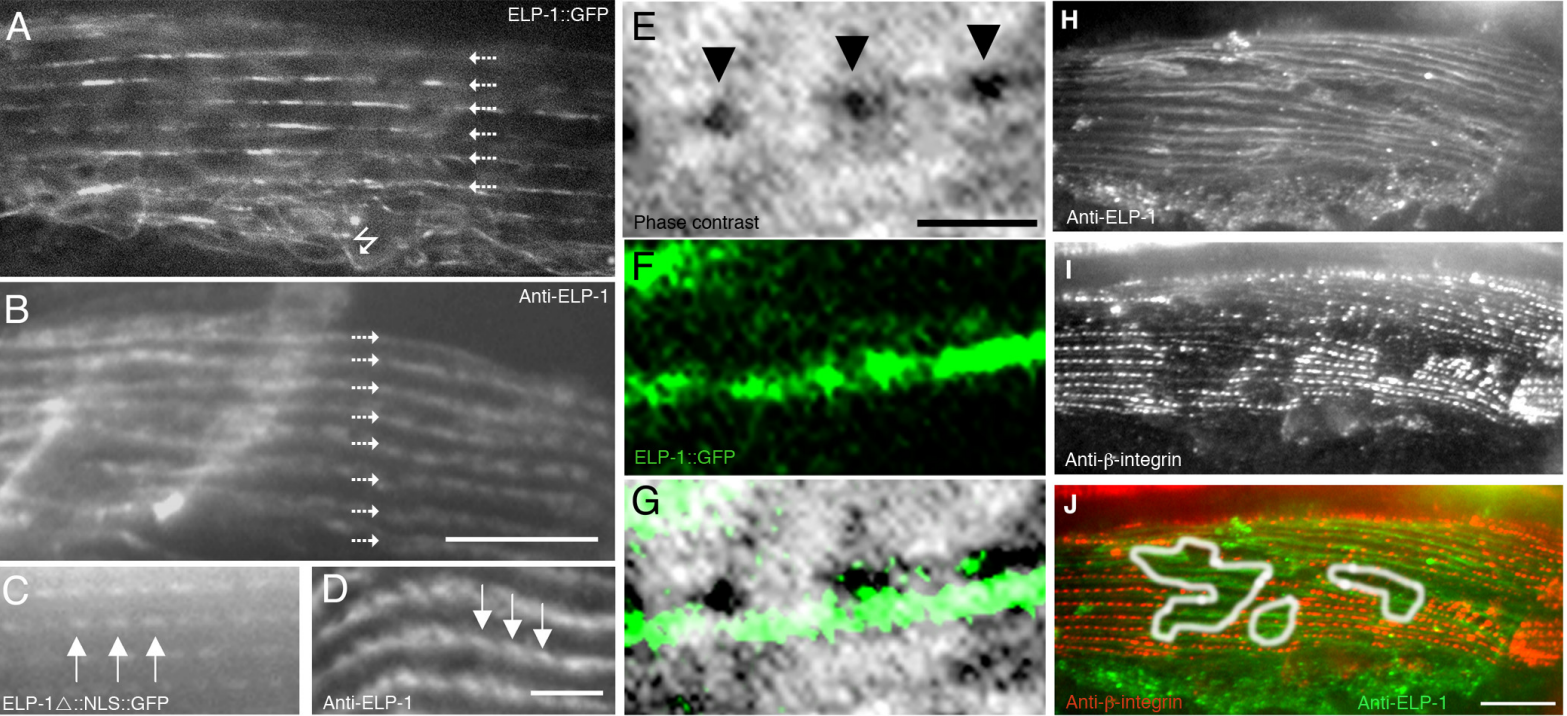
**ELP-1 localizes to body wall muscle in hermaphrodites.** (A) Near the muscle cell membrane, ELP-1::GFP is prominent in lines (small arrows) oriented with the longitudinal axis of the cell. ELP-1::GFP is also associated with an array of fluorescent filaments (jagged arrow). (B) Affinity-purified antibodies against ELP-1 also stain a linear array near the cell surface. Bar represents 10 μm. Within these "lines" there often is a repeating unit of fluorescence that is apparent in both transgenic animals (C) and in antibody-stained animals (D). Bar represents 2.5 μm. (E) Dense bodies (arrowheads) are shown at the muscle cell surface by phase contrast microscopy. ELP-1::GFP expression was examined via fluorescence light microscopy (F) and the two images taken at the same focal plane were overlaid in Panel G. Bar represents 1 μm. Muscle cells that were double-stained with antibodies against ELP-1 (H, J) and PAT-3/β-integrin (MH25) (I, and J) show overlapping staining patterns. The integrin puncta are superimposed upon the more linear staining pattern of ELP-1. When the membrane is removed by the freeze-cracking procedure (outlined in white in panel J), integrin is lost and the ELP-1 antigen remains. Bar represents 10 μm.

Hermaphrodites were examined by fluorescent microscopy with the optical axis perpendicular to the longitudinal axis of a body wall muscle cell. Affinity-purified antibodies against ELP-1 and the full-length ELP-1::GFP fusion protein localized to thin linear rows near the cell surface that were not quite parallel to the long axis of the muscle cell (Figure 6A, B). At higher resolution there was a periodicity to these lines that approximated the distance between the dense body adhesion sites (Figure 6C, D).

Body wall muscle cells are anchored along their length to the basement membrane by the dense bodies, finger-like projections analogous to the vertebrate Z-lines. Anchorage of actin in the myofilament lattice to the dense bodies is necessary for force transduction in body wall muscle. Dense body puncta are distinctive because they are aligned in a row that runs at a 6-degree pitch from the longitudinal axis of the muscle cell [25].

In Figure 6E, individual dense bodies are shown as phase-dark puncta. The fluorescent image of ELP-1::GFP was examined at the same focal plane (Figure 6F) and these micrographs were overlaid and shown in Figure 6G. The ELP-1::GFP fluorescence slightly overlaps the edges of the dense bodies but is not superimposable with the dense bodies at this angle. These results indicate that ELP-1 is unlikely to be a component of the dense bodies.

To further examine the association of ELP-1 with dense bodies, we double-stained muscle cells with anti-ELP-1 antibodies and a monoclonal antibody MH25 against β integrin (PAT-3), an integral membrane component that anchors the dense bodies to the sarcolemma. Figure 6 H-J shows that integrin and ELP-1 have overlapping staining patterns at this level of resolution. Because of the relatively impenetrable cuticle that surrounds the nematode, these worms were frozen and cracked open prior to antibody staining. Occasionally a portion of the muscle cell membrane was removed carrying along its complement of dense bodies (see white outline in Figure 6J). In areas lacking dense bodies, ELP-1::GFP was sometimes lost. However most of the ELP-1::GFP was retained in a linear pattern. These results indicate that the membrane and dense bodies can be separated from the ELP-1::GFP.

### ELP-1 is associated with microtubules

**Figure 7:**
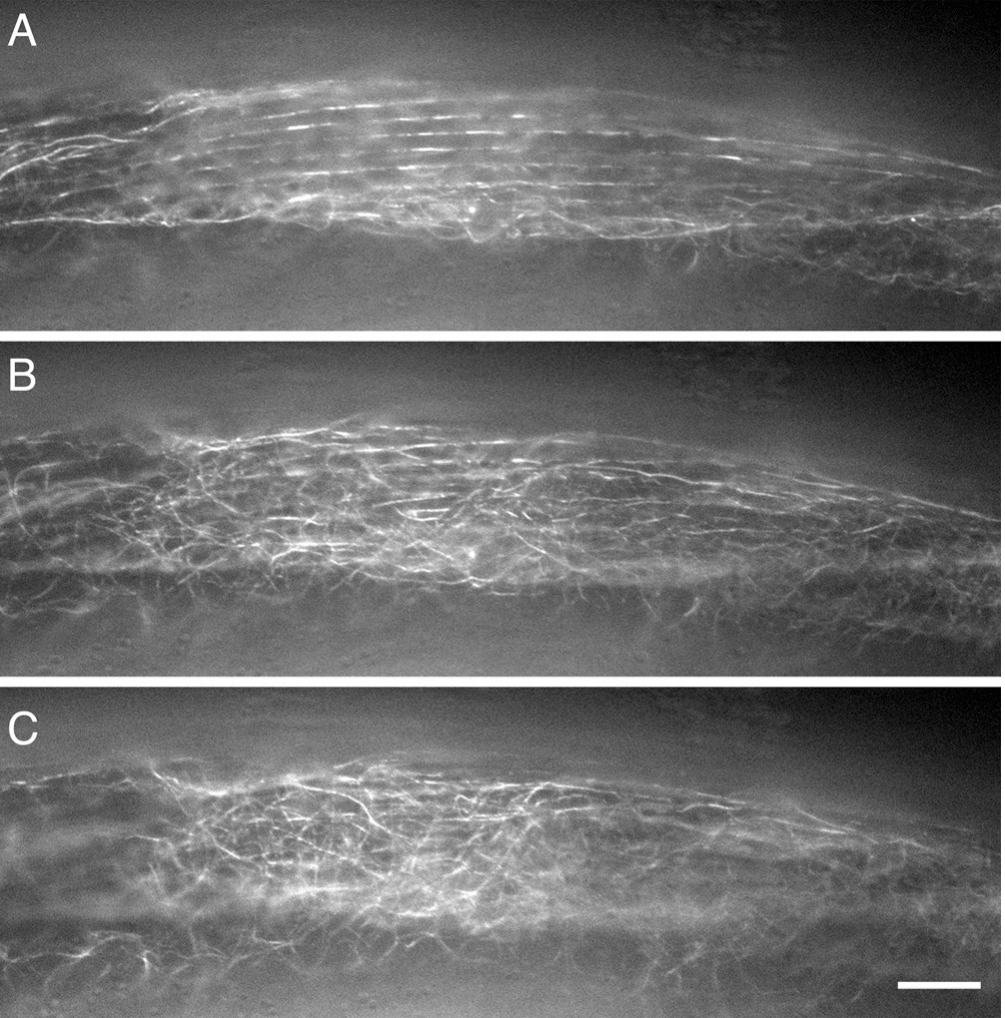
**Analysis of subcellular filaments in a single body wall muscle cell of a hermaphrodite.** These three panels (A-C) were part of a Z-series of images that were taken every 0.2 μm from the muscle cell membrane towards the interior of the muscle cell. The first image in Panel A was taken approximately 0.8 μm from the cell surface (4 sections down from the surface). At this focal plane, ELP-1::GFP is associated with oblique linear striations overlap with the anti-β integrin (MH25) staining pattern shown in Figure 6. In successive focal planes (B and C) the filaments are no longer in linear arrays and have branched off into a criss-crossing array. Bar represents 10 μm.

**Figure 8:**
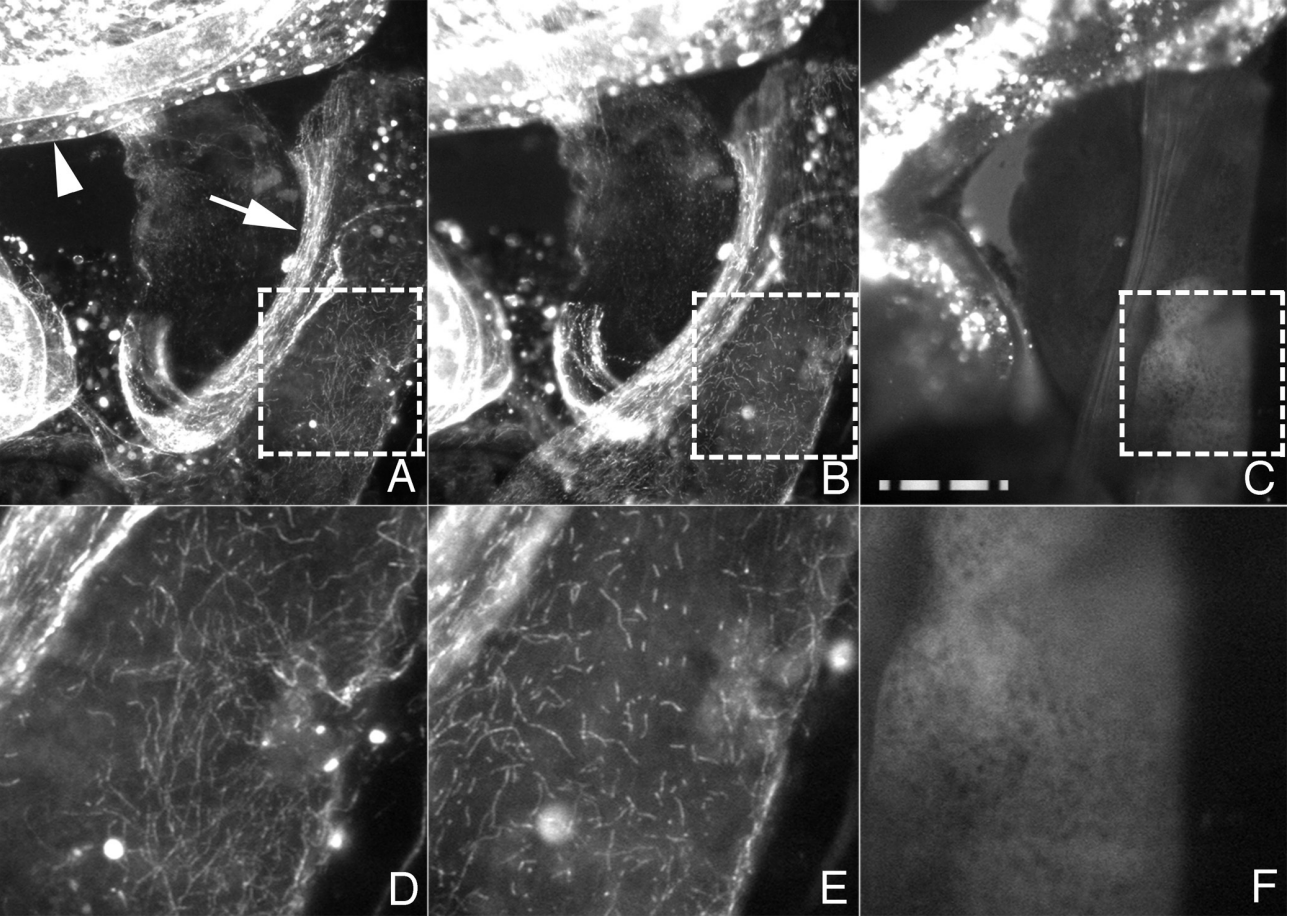
**ELP-1::GFP is associated with microtubules.** ELP-1::GFP associates with fluorescent filaments in a squashed-worm preparation (A & D). A 1-minute treatment with levamisole (25 mM) and nocodazole (10 μM) shortens filaments (B & E). Longer treatments (2 minutes) eliminate fluorescent filaments (C & F). The boxes outlined with the dotted lines in A, B and C are enlarged in panels D, E, and F. Filaments in A & D were stable for at least 15 minutes of observation. The bright punctate fluorescence in the upper left of each panel is due to the autofluorescence of the gut granules (arrowhead). The spacing on the magnification bar is 10 μm. Similar results were observed in a total of three squashed-worm preparations.

Although the truncated construct and the full-length construct were expressed in the same cells and tissues, only the full length ELP-1::GFP construct localized to an elaborate array of fluorescent filaments. These filaments, which resembled microtubules, were obvious in the larger cells of the worm including the body wall muscle (Figure 7) and intestine (Figure 8). An obliquely striated fluorescent pattern emerges approximately 0.8 μm apically from the base of the dense body. Deeper within the muscle body the filaments appear to separate from the linear track and branch out into the cytoplasm.

To test the idea that the filaments decorated by ELP-1::GFP were microtubules, we treated worms with the microtubule inhibitor, nocodazole. Because the worm cuticle is an effective barrier to nocodazole, we gently pressed worms between a slide and coverslip to extrude their intestines. This process was done in the presence of levamisole, an acetylcholine agonist, which was used to paralyze the worms for microscopy. In Figure 8, the worm was flattened and the intestines were gently extruded. The filaments in the extruded layer were stable for 30 minutes or more in the absence of nocodazole. The decorated filaments began to degrade within seconds and were entirely de-polymerized after two minutes in nocodazole. When the fluorescence disappeared the intestinal fragments noticeably flattened and the remnants of the worm contracted. This result indicates that ELP-1::GFP associates with microtubules *in situ*.

**Figure 9:**
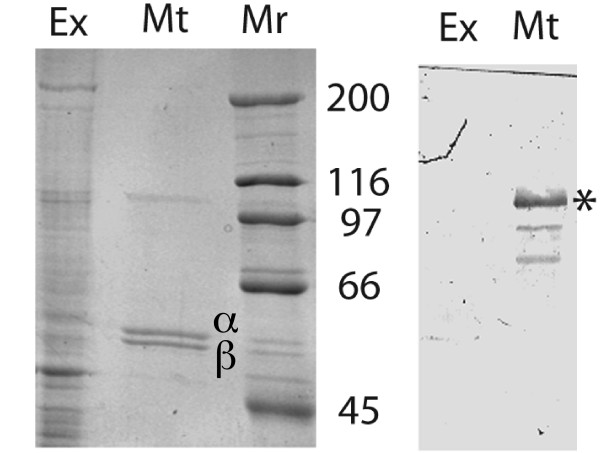
**ELP-1 antigens co-pellet with paclitaxel-stabilized *C. elegans *microtubules.** Paclitaxel-stabilized microtubules (Mt) were prepared from a mixed-stage worm extract (Ex) as described in the Materials and Methods. The panel on the left shows the protein complexity in a *C. elegans *extract (Ex, 25,000 *g *supernatant) and in a Paclitaxel-stabilized microtubule preparation (Mt, 25,000 *g *pellet). An ~100 kDa polypeptide and α and β tubulin are the most prominent proteins visualized in the microtubule preparation. Proteins were separated on an 8% acrylamide mini-gel (left panel) and probed with affinity-purified anti-ELP-1 antibodies on a nitrocellulose blot (right panel). Asterisk is next to the ~100 kDa band that cross-reacts with the anti-ELP-1 antibodies. Molecular masses × 10^-3 ^(Mr) are shown to the left. Approximately 15 μg of protein was loaded in each lane of a 7% polyacrylamide SDS-page mini-gel.

Finally, we show that ELP-1 was enriched in preparations of paclitaxel-stabilized microtubules *in vitro*. Paclitaxel-stabilized microtubules were prepared from a mixed-stage worm preparation and examined by means of Western blotting with an affinity-purified antisera against a bacterially expressed ELP-1 fusion protein (Figure 9). An ELP-1-reacting band co-purified with microtubules and migrated at ~100 kDa, a mass that corresponded to the Genefinder™ predictions for the larger ELP-1a polypeptide. Two smaller and less abundant proteins that cross-react with the ELP-1 antibodies also co-purified with microtubules. These may be isoforms generated by alternative splicing (i.e. ELP-1b) or proteolytic fragments of ELP-1a. This experiment and the one described above showed that ELP-1 is microtubule-associated both *in situ *and *in vivo*. Whether ELP-1 binds directly to tubulin remains to be determined.

### ELP-1 is needed for normal touch-sensitivity

**Figure 10:**
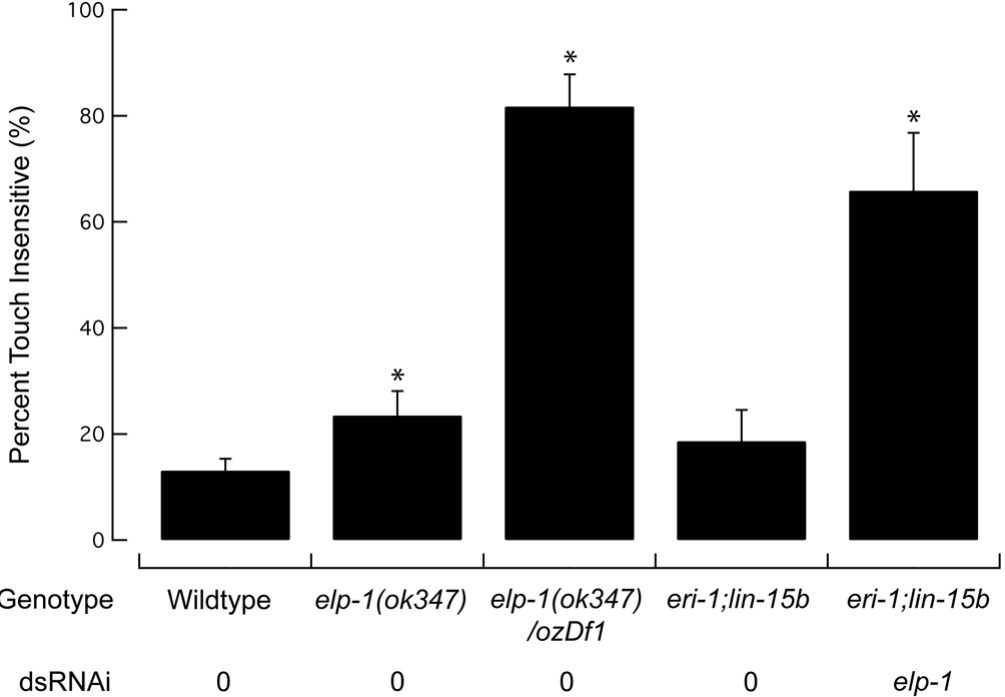
**ELP-1 is needed for normal touch sensation.** Bars are mean ± s.d. for at least three independent assays of 30–50 animals each. RNAi knockdown of *elp-1 *in the *lin-15b;eri-1 *background induces a stronger touch-insensitive phenotype (66 ± 11%, mean ± s.d.) than does partial deletion of *elp-1(ok347) *(24 ± 5%) and is similar to the defect found in *ok347/ozDf1 *heterozygotes (82 ± 6%). Percentage of touch-insensitive animals for the remaining genotypes and RNAi treatments are as follows: wild-type (12 ± 0%) and *lin-15b;eri-1 *without RNAi (19 ± 6%). Tests were conducted blind to genotype or RNAi treatment. *P < 0.001 Fisher's Exact test.

Based on its expression in the TRNs and its intimate association with microtubules, we hypothesized that ELP-1 plays a role in gentle-touch sensation. We tested this idea by measuring touch-sensitivity in mutants carrying defects in the *elp-1 *gene and in animals treated with RNAi directed against ELP-1. We found that the *ok347 *allele significantly increases the proportion of touch-insensitive animals (Figure 10). This is unlikely to be the null phenotype, however, since *ok347 *mutants retain two transcripts predicted to encode nearly full-length ELP-1 proteins containing both the HELP and WD repeat domains (Figure 2E). Thus, *ok347 *is predicted to be a partial loss-of-function allele. Consistent with this idea, the proportion of touch-insensitive animals was dramatically increased when the *elp-1(ok347) *allele was placed *in trans *to a deficiency that covers the *elp-1 *gene (*ozDf1*) and when ELP-1 expression was decreased by feeding RNAi-expressing bacteria (Figure 10). These results demonstrate that wild-type ELP-1 is needed for normal touch sensitivity in *C. elegans *and indicate that EMAP-like proteins could play a critical role in sensory mechanotransmission.

## Discussion

EMAP and EMAP-like proteins are conserved in nematodes, insects, echinoderms and vertebrates [8,26-31]. Most members of the superfamily associate with microtubules [8,9,11-13] and several are implicated in regulating microtubule stability during mitosis [8,9,11,27]. Their expression is not limited to dividing cells, however. The function of EMAP-like proteins in post-mitotic cells such as neurons is poorly understood. To learn more, we combined molecular and behavioural genetics with cell biological approaches to analyze ELP-1, the sole EMAP-like protein in the *C. elegans *genome. Because all 959 somatic cells in each adult hermaphrodite are post-mitotic, this analysis offers new insight into the cell biology and physiology of ELP-1 in post-mitotic cells and tissues.

We show here that ELP-1 is prominently expressed in cells that make productive interactions with the cuticle. In males and hermaphrodites, these include mechanoreceptor neurons such as the TRNs and diverse muscle cell types. In males, ELP-1 is found in the hypodermal cells and neurons of the male sensory rays. Additionally, ELP-1 is found in the cell bodies, throughout the neuronal processes, and in the ciliated endings of all six IL1 neurons. The IL1 neurons are putative mechanoreceptor neurons needed for wild-type foraging movements and for sensing touch applied to the nose [32].

ELP-1 is a promising candidate for regulating microtubule function in mechanoreceptor cells. For example, the TRNs are unique in that their sensory processes contain 15-protofilament microtubules rather than 11-protofilament microtubules observed in the majority of *C. elegans *cells [33]. Approximately 450 of these large-diameter microtubules are bundled together and constrained to move as a single structure by distinct 10 nm filaments [19,32,34]. The bundles are also linked to the plasma membrane through a series of 14 nm filaments [19]. ELP-1 is an attractive candidate to bundle microtubules. In addition, the HELP motif, rich in hydrophobic amino acids, could link the bundles to the plasma membrane. In either scenario, ELP-1 would contribute to TRN function by amplifying the forces generated at the cuticle. Consistent with this idea, reducing ELP-1 levels decreased touch-sensitivity.

In addition to mechanoreceptor neurons, ELP-1 is expressed in cells that utilize cadherin-based apical junctions and or fibrous organelles for cell adhesion and attachment to the basal lamina. These include the cells of the intestine, body wall, vulval, and male-specific sex muscles, hypodermis and seam cells [35]. The hemidesomosome-like fibrous organelles, formed by the hypodermis, transmit cuticle deformation to the touch receptor neurons and muscle tension to the cuticle [36,37]. Microtubules are interspersed with actin filaments near these fibrous organelles, however it is not known how they might be anchored to the plasma membrane [35]. We speculate that ELP-1 is involved in the anchoring or bundling of microtubules to the plasma membrane and perhaps the transmission of forces therein.

**Figure 11:**
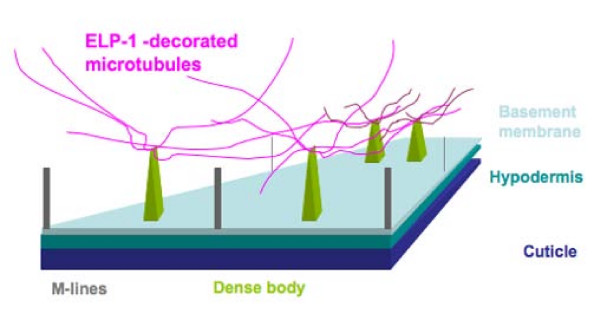
**Three-dimensional sketch illustrating the relationship of ELP-1-associated microtubules and the dense bodies (green).** A transverse section through a single body wall muscle cell is shown. The thin filaments, which are attached to the dense bodies, and the thick filaments attached to the M-lines, have been eliminated from the sketch for clarity. ELP-1 associated microtubules (magenta) are associated with the apical portion of the dense body. Neighbouring dense bodies are linked by microtubules giving rise to the linear staining patterns in Figure 6. The microtubules extend into the cytosol that contains the nucleus, mitochondria, and other membranes (not shown).

ELP-1 may also be involved in force transmission and synapse formation in body wall muscle. *C. elegans *has four longitudinal muscle quadrants that are anchored to the cuticle through the hypodermis in order to generate locomotion [38]. Specifically, the myofibrils are anchored to the extracellular matrix by dense bodies, structures similar to integrin-based, vertebrate focal adhesions [39]. In addition, muscle arms project towards motor axons in the nerve cord to form the postsynaptic element of the neuromuscular junction [32,40]. ELP-1 is found in the muscle arms and at the dense bodies of body wall muscle cells. In muscle cells ELP-1 may function to link the microtubules to the adhesion sites, regulate the assembly and disassembly of microtubules, or even deliver or remove a regulatory molecule to adhesion sites (Figure 11).

It is intriguing that ELP-1 is associated with adhesion sites similar to mammalian focal adhesions. It has been known for some time that microtubules can locate or target focal adhesions [41] and that depolymerization of microtubules enhance actomyosin-based cell contractility [42]. The increase in cell contractility is mediated by GEF-H1 [43,44], a microtubule-associated guanine nucleotide exchange factor that activates the small G-protein, RhoA [45]. Upon microtubule depolymerization, GEF H1 is released and activates the Rho-associated kinase (ROCK) that phosphorylates the myosin regulatory light chain (MLC) resulting in increased contractility [46,47]. Whether a similar pathway is functional in *C. elegans *muscle remains to be determined.

## Conclusion

In *C. elegans*, ELP-1 is the sole member of a highly conserved family of metazoan microtubule-binding proteins. In adults, ELP-1 is expressed in post-mitotic cells that make productive interactions with the cuticle such as body wall muscle and mechanoreceptor neurons, suggesting a role in force generation and sensing. In support of this idea, disrupting ELP-1 expression by mutation and RNAi renders animals insensitive to gentle body touch. This study is a critical first step toward elucidating the function of EMAP-like proteins in post-mitotic cells, including many mechanoreceptor neurons.

## Methods

### Reagents

Stock solutions of levamisole, DiD (1,1'-dioctadecyl-3, 3,3', 3'-tetramethylindodicarbocyanine, 4-chlorobenzenesulfonate salt, Invitrogen-Molecular Probes, Carlsbad, CA), paclitaxel (Taxol™, Merck-Calbiochem, San Diego, CA), and nocodazole were stored at -20°C and prepared as follows (solvent, concentration): levamisole (M9 buffer, 100 mM); DiD (1 mg/ml, dimethyl formamide); paclitaxel (10 mM, dimethylsulfoxide); and, nocodazole (10 mM, dimethylsulfoxide).

### *C. elegans *strains and culture

Wild-type worms (N2, Bristol) were grown on NGM plates seeded with the *E. coli *strain OP50 at 20°C, unless otherwise indicated [48,49]. MT8189 *lin-15(n765ts) *X, KP3948 *eri-1(mg366) *IV; *lin-15B(n744) *X, TU38 *deg-1(u38) X*, RB639 *elp-1(ok347) *V, and BS518 *ozDf1/sdc-3*(y52y180) *unc-76*(e911)V animals were supplied by the *Caenorhabditis *Genetics Center, Minneapolis. Genotypes of new strains generated to examine tissue specific expression include *lkEx4*[*P_elp-1_::elp-1(exon Δ11–16)::nls::gfp *(pKA99-1); *rol-6(su1006)*] (KA15-17), *(lin-15(n765)*; *lkEx1*[*P_elp-1_::elp-1::gfp (pKA99-2); lin-15(+)]) *(KA6-8), *(lin-15(n765)*; lkEx3[*P_elp-1_::gfp *(pKA99-3); *lin-15(+)])*, (KA14) and *deg-1(u38)X;lkEx4*[*P_elp-1_::elp-1::gfp *(pKA99-2); *rol-6(su1006)] *(KA37).

The *elp-1(ok347) *strain, RB639, was obtained from the *C. elegans *Gene Knockout Consortium at the Oklahoma Medical Research Foundation (Oklahoma City, OK), out-crossed four times and the exact location of the deletion was confirmed by sequencing a PCR product that spanned the deletion (Figure 2D).

### Non-complementation of *elp-1(ok347) *allele and *ozDf1 *deficiency

To test whether *elp-1(ok347) *was a null or hypomorphic allele of *elp-1*, the *elp-1(ok347) *allele was placed *in trans *to *ozDf1*. The deficiency *ozDf1 *maps to linkage group V (left end: 10.318; right end 25.1193) and deletes *dpy-21*, *pro-3*, *srf-4*, and *unc-51 *[50] and covers *elp-1*. The absence of *elp-1 *in strain BS518 was confirmed via PCR.

### Western blotting and ELP-1 antibodies

A 2595-bp cDNA clone for *elp-1b *(yk209e10) was kindly provided by Y. Kohara at the DNA Data Bank of Japan . The insert was sub-cloned into a pET14b expression vector (Novagen, Madison, WI). The 6-His-tagged ELP-1b (aa1-864) was expressed in *E. coli *(BL21(DE3)) and purified under denaturing conditions via chromatography on an iminodiacetic acid Sepharose column [51]. Antibodies were generated in rabbits against the purified 6-His-tagged ELP-1b immunogen and affinity-purified as specified [52].

Proteins were separated on 8% acrylamide mini-gels, transferred to nitrocellulose, probed with affinity-purified anti-ELP-1 antibodies (1:1000), and visualized with alkaline phosphatase-conjugated secondary antibodies and chemiluminescence.

### Tissue-specific expression

Vectors that express ELP-1 fused to GFP were created from the Fire Lab Vectors  by PCR amplification or restriction digestion of the genomic cosmid, *F38A6.2*. Standard molecular techniques were used and all PCR products were cloned and sequenced to be certain that no errors were introduced during amplification. The *P_elp-1_::elp-1(Δ11–16)::nls::gfp *construct (pKA99-1) was generated by ligating a SphI-BamHI genomic fragment that contains 4 kb of the *elp-1 *5'UTR region (*P_elp-1_*) and 3 kb of the *elp-1 *gene (exons 1–10) into the pPD95.67 vector upstream of a nuclear localization sequence (NLS) and the *gfp *gene. The construct expresses a truncated ELP-1 protein fused to GFP with an NLS under the endogenous *elp-1 *promoter. The *P_elp-1_::elp-1::gfp *construct (pKA99-2) contains 9 kb of the *elp-1 *gene with the endogenous *elp-1 *promoter (*P_elp-1_*) inserted into the SphI-XbaI site in the pPD95.75 vector and expresses the full-length ELP-1 protein (exons 1–16) fused to GFP. *elp-1 *was amplified from the F38A6 cosmid with the Expand Long Template PCR system (Roche-Boehringer Mannheim; Alameda, CA) with the primers, EMAPUSGFP1-5', corresponding to sequence upstream of the *SphI *genomic restriction site (5'-AACACCGAACTTGATGAAATATTCGGTGCAAC-3') and EMAPSTP2GFP2-3' (5'CCTCTCTAGATCCGCTTCCAGGCACCATTCAAAAACCGAATTATCAG-3'), corresponding to the sequence at the 3' end of the coding region of the gene, and substituting an *XbaI *restriction site for the stop codon. The *P_elp-1_::gfp construct *(pKA99-3) expresses GFP under the control of the 4 kb *elp-1 *promoter region. For this construct the promoter region was amplified with primer, EMAPUSGFP1-5' and primer, EMAPSTRTGFP1-3' (5'-CGGGGATCCTCCATTTTTTTGAAGAATTTTTGCAAATTTTCTCCTGCAAC-3'), corresponding to sequence at the start site of the gene, with a *BamHI *tag (GGGATCC) used to ligate into the pPD95.75 vector.

Transgenic strains carrying each of these extrachromosomal arrays (200 ng/μl) were established with *rol-6(su1006) *(100 ng/μl) as a transformation marker in N2 worms or with the rescue reporter *lin-15(+) *(100 ng/μl) injected into a *lin-15(n765ts) *mutant strain. Three independent array-bearing lines were isolated for each array with each line showing the same tissue expression pattern.

### DiD dye-filling protocol

The inner labial 2 (IL2) sensory neurons were labelled with 10 μg/ml DiD in M9 buffer containing 50 mM calcium acetate (personal communication from Elizabeth Ryder, Worcester Polytechnic Institute). After incubation for one hour, the animals were washed twice with dH_2_O and put onto an OP50 feeding plate for 1 hr to allow the excess DiD to pass through the intestines. Worms were washed off the plate with M9 and placed on 2% agar pads in a drop of M9 buffer containing 10 mM sodium azide, covered with a coverslip, and examined via fluorescence light microscopy.

### Nocodazole treatment

Microtubules were disrupted in adult worms via a protocol adapted from a previous study of the microtubule binding protein ZYG-9 [53]. ELP-1::GFP expressing worms were gently flattened between a coverslip and slide to extrude a portion of the intestine. Squashed worms were perfused three times with a small volume (10 μl) of M9 buffer containing levamisole (25 mM) and observed for ~15 minutes. We disrupted microtubules in this preparation using nocodazole, an antagonist of microtubule polymerization. Nocodazole (10 μM) was applied by superfusing squashed worms with 10 μl of solution.

### Immunostaining

Immunofluorescent staining of worms was carried out following the methods of Miller and Shakes (1995) [54]. A mixed population of worms was freeze-cracked by immersion in liquid nitrogen, fixed in ice-cold methanol (15 min) and in ice-cold acetone (10 min). Animals were washed twice in PBT [PBS containing 0.1% Triton-X (v/v) and 0.1% BSA (w/v)] and stained with primary antibodies overnight at 4°C. The primary antibodies used were the affinity-purified anti-ELP-1 antibodies (diluted 1:50 in PBST with BSA) and a mouse monoclonal anti-PAT-3 antibody MH25 (a gift from Michelle Hresko, Washington University School of Medicine, St. Louis, [38]) (diluted 1:250 in PBST). The worms were washed three times in PBST and then incubated with the secondary antibodies: Cy2-conjugated donkey anti-rabbit [1:250] or Cy3-conjugated donkey anti-mouse [1:250] in PBT buffer overnight at 4°C. Lastly, the slides were washed three times in 1× PBT and mounted in PBT buffer.

### Light Microscopy

*C. elegans *were examined by means of bright field, differential interference contrast (DIC), and fluorescence microscopy [49]. Worms were anesthetized with a drop of 1% 1-phenoxy-2-propanol in M9 buffer and placed on 2% agar pads on glass slides. The highest resolution images were taken with a 60× 1.4 NA Plan-Apochromat Nikon objective and captured with a Hamamatsu Orca-ER camera (Hamamatsu City, Japan) driven by the OpenLab software V3 (Improvision, Lexington, MA). Individual files were compiled and contrast-balanced when necessary using Adobe PhotoShop. All the images for each dataset were treated the same.

### RNAi-mediated gene knockdown

Recently hatched larvae of the RNAi-hypersensitive mutant, *lin-15b;eri-1*, were fed RNAi-expressing bacteria from the Ahringer RNAi feeding library [55].

### Touch assay

Young adult animals were alternately touched on the anterior and posterior region of the body a total of ten times with an eyebrow hair glued to a toothpick [56]. Animals that failed to respond to all ten touches were considered touch-insensitive. Three independent assays of 30–50 animals were tested. Tests were conducted blind to RNAi treatment or to genotype.

## Authors' contributions

JLH carried out the molecular genetics, generated the expression data and contributed to drafting the manuscript. GPH carried out the dye-filling assays and contributed to the identification of the mechanoreceptor neurons. MJB and EAL contributed to study design and drafting the manuscript. JGC carried out behavioural studies and contributed to drafting the manuscript. MBG participated in study design and coordination, and helped to draft the manuscript. KAS conceived the study, carried out the microtubule assays, advised on all experiments, and drafted the manuscript from the dissertation and thesis of JLH and GPH, respectively. All authors have read and approve the manuscript.

## References

[B1] Basu R, Chang F (2007). Shaping the actin cytoskeleton using microtubule tips. Curr Opin Cell Biol.

[B2] Kwok BH, Kapoor TM (2007). Microtubule flux: drivers wanted. Curr Opin Cell Biol.

[B3] Siegrist SE, Doe CQ (2007). Microtubule-induced cortical cell polarity. Genes Dev.

[B4] Akhmanova A, Hoogenraad CC (2005). Microtubule plus-end-tracking proteins: mechanisms and functions. Curr Opin Cell Biol.

[B5] Caviston JP, Holzbaur EL (2006). Microtubule motors at the intersection of trafficking and transport. Trends Cell Biol.

[B6] Howard J, Hyman AA (2007). Microtubule polymerases and depolymerases. Curr Opin Cell Biol.

[B7] Suprenant KA, Daggett MA (1995). Sea urchin microtubules. Current Topics in Developmental Biology.

[B8] Tegha-Dunghu J, Neumann B, Reber S, Krause R, Erfle H, Walter T, Held M, Rogers P, Hupfeld K, Ruppert T (2008). EML3 is a nuclear microtubule-binding protein required for the correct alignment of chromosomes in metaphase. J Cell Sci.

[B9] Hamill DR, Howell B, Cassimeris L, Suprenant KA (1998). Purification of a WD repeat protein, EMAP, that promotes microtubule dynamics through an inhibition of rescue. J Biol Chem.

[B10] Eichenmuller B, Ahrens DP, Li Q, Suprenant KA (2001). Saturable binding of the echinoderm microtubule-associated protein (EMAP) on microtubules, but not filamentous actin or vimentin filaments. Cell Motil Cytoskeleton.

[B11] Eichenmuller B, Everley P, Palange J, Lepley D, Suprenant KA (2002). The human EMAP-like protein-70 (ELP70) is a microtubule destabilizer that localizes to the mitotic apparatus. J Biol Chem.

[B12] Pollmann M, Parwaresch R, Adam-Klages S, Kruse ML, Buck F, Heidebrecht HJ (2006). Human EML4, a novel member of the EMAP family, is essential for microtubule formation. Exp Cell Res.

[B13] Houtman SH, Rutteman M, De Zeeuw CI, French PJ (2007). Echinoderm microtubule-associated protein like protein 4, a member of the echinoderm microtubule-associated protein family, stabilizes microtubules. Neuroscience.

[B14] De Keersmaecker K, Graux C, Odero MD, Mentens N, Somers R, Maertens J, Wlodarska I, Vandenberghe P, Hagemeijer A, Marynen P (2005). Fusion of EML1 to ABL1 in T-cell acute lymphoblastic leukemia with cryptic t(9;14)(q34;q32). Blood.

[B15] Inamura K, Takeuchi K, Togashi Y, Nomura K, Ninomiya H, Okui M, Satoh Y, Okumura S, Nakagawa K, Soda M (2008). EML4-ALK fusion is linked to histological characteristics in a subset of lung cancers. J Thorac Oncol.

[B16] Soda M, Choi YL, Enomoto M, Takada S, Yamashita Y, Ishikawa S, Fujiwara S, Watanabe H, Kurashina K, Hatanaka H (2007). Identification of the transforming EML4-ALK fusion gene in non-small-cell lung cancer. Nature.

[B17] Suprenant KA, Tuxhorn JA, Daggett MA, Ahrens DP, Hostetler A, Palange JM, VanWinkle CE, Livingston BT (2000). Conservation of the WD-repeat, microtubule-binding protein, EMAP, in sea urchins, humans, and the nematode C. elegans. Dev Genes Evol.

[B18] Ernstrom GG, Chalfie M (2002). Genetics of sensory mechanotransduction. Annu Rev Genet.

[B19] Cueva JG, Mulholland A, Goodman MB (2007). Nanoscale organization of the MEC-4 DEG/ENaC sensory mechanotransduction channel in Caenorhabditis elegans touch receptor neurons. J Neurosci.

[B20] Perkins LA, Hedgecock EM, Thomson JN, Culotti JG (1986). Mutant sensory cilia in the nematode Caenorhabditis elegans. Dev Biol.

[B21] Chalfie M, Wolinsky E (1990). The identification and suppression of inherited neurodegeneration in Caenorhabditis elegans. Nature.

[B22] Hedgecock EM, Culotti JG, Hall DH (1990). The unc-5, unc-6, and unc-40 genes guide circumferential migrations of pioneer axons and mesodermal cells on the epidermis in C. elegans. Neuron.

[B23] Hall DH, Hedgecock EM (1991). Kinesin-related gene unc-104 is required for axonal transport of synaptic vesicles in C. elegans. Cell.

[B24] Sulston JE, Albertson DG, Thomson JN (1980). The Caenorhabditis elegans male: postembryonic development of nongonadal structures. Dev Biol.

[B25] Moerman DG, Williams BD (2006). Sarcomere assembly in C. elegans muscle. WormBook.

[B26] O'Connor V, Houtman SH, De Zeeuw CI, Bliss TV, French PJ (2004). Eml5, a novel WD40 domain protein expressed in rat brain. Gene.

[B27] Heidebrecht HJ, Buck F, Pollmann M, Siebert R, Parwaresch R (2000). Cloning and localization of C2orf2(ropp120), a previously unknown WD repeat protein. Genomics.

[B28] Eudy JD, Ma-Edmonds M, Yao SF, Talmadge CB, Kelley PM, Weston MD, Kimberling WJ, Sumegi J (1997). Isolation of a novel human homologue of the gene coding for echinoderm microtubule-associated protein (EMAP) from the Usher syndrome type 1a locus at 14q32. Genomics.

[B29] Lepley DM, Palange JM, Suprenant KA (1999). Sequence and expression patterns of a human EMAP-related protein-2 (HuEMAP-2). Gene.

[B30] Li Q, Callaghan M, Suprenant KA (1998). The 77-kDa echinoderm microtubule-associated protein (EMAP) shares epitopes with the mammalian brain MAPs, MAP-2 and tau. Biochem Biophys Res Commun.

[B31] Li Q, Suprenant KA (1994). Molecular characterization of the 77-kDa echinoderm microtubule-associated protein. Homology to the beta-transducin family. J Biol Chem.

[B32] White JG, Southgate E, Thomson JN, Brenner S (1986). The structure of the nervous system of the nematode C. elegans. Philosophical Transactions of the Royal Society of London-Series B: Biological Sciences.

[B33] Chalfie M, Thomson JN (1982). Structural and functional diversity in the neuronal microtubules of Caenorhabditis elegans. J Cell Biol.

[B34] Chalfie M, Thomson JN (1979). Organization of neuronal microtubules in the nematode Caenorhabditis elegans. J Cell Biol.

[B35] Labouesse M (2006). Epithelial junctions and attachments. WormBook.

[B36] Hresko MC, Schriefer LA, Shrimankar P, Waterston RH (1999). Myotactin, a novel hypodermal protein involved in muscle-cell adhesion in Caenorhabditis elegans. J Cell Biol.

[B37] Francis R, Waterston RH (1991). Muscle cell attachment in Caenorhabditis elegans. J Cell Biol.

[B38] Francis GR, Waterston RH (1985). Muscle organization in Caenorhabditis elegans: localization of proteins implicated in thin filament attachment and I-band organization. J Cell Biol.

[B39] Cox EA, Hardin J (2004). Sticky worms: adhesion complexes in C. elegans. J Cell Sci.

[B40] Dixon SJ, Roy PJ (2005). Muscle arm development in Caenorhabditis elegans. Development.

[B41] Small JV, Geiger B, Kaverina I, Bershadsky A (2002). How do microtubules guide migrating cells?. Nat Rev Mol Cell Biol.

[B42] Danowski BA (1989). Fibroblast contractility and actin organization are stimulated by microtubule inhibitors. J Cell Sci.

[B43] Krendel M, Zenke FT, Bokoch GM (2002). Nucleotide exchange factor GEF-H1 mediates cross-talk between microtubules and the actin cytoskeleton. Nat Cell Biol.

[B44] Birukova AA, Birukov KG, Adyshev D, Usatyuk P, Natarajan V, Garcia JG, Verin AD (2005). Involvement of microtubules and Rho pathway in TGF-beta1-induced lung vascular barrier dysfunction. J Cell Physiol.

[B45] Hall A (1998). Rho GTPases and the actin cytoskeleton. Science.

[B46] Chang YC, Nalbant P, Birkenfeld J, Chang ZF, Bokoch GM (2008). GEF-H1 Couples Nocodazole-induced Microtubule Disassembly to Cell Contractility via RhoA. Mol Biol Cell.

[B47] Kolodney MS, Elson EL (1995). Contraction due to microtubule disruption is associated with increased phosphorylation of myosin regulatory light chain. Proc Natl Acad Sci USA.

[B48] Brenner S (1974). The genetics of Caenorhabditis elegans. Genetics.

[B49] Sulston JE, Hodgkin J (1988). Methods. The Nematode Caenorhabditis elegans.

[B50] Clifford R, Lee MH, Nayak S, Ohmachi M, Giorgini F, Schedl T (2000). FOG-2, a novel F-box containing protein, associates with the GLD-1 RNA binding protein and directs male sex determination in the C. elegans hermaphrodite germline. Development.

[B51] Picking WL, Mertz JA, Marquart ME, Picking WD (1996). Cloning, expression, and affinity purification of recombinant Shigella flexneri invasion plasmid antigens IpaB and IpaC. Protein Expr Purif.

[B52] Harlow E, Lane D (1988). Antibodies: A Laboratory Manual. Cold Spring Harbor Laboratory.

[B53] Matthews LR, Carter P, Thierry-Mieg D, Kemphues K (1998). ZYG-9, a Caenorhabditis elegans protein required for microtubule organization and function, is a component of meiotic and mitotic spindle poles. J Cell Biol.

[B54] Miller DM, Shakes DC (1995). Immunofluorescence microscopy. Methods Cell Biol.

[B55] Ahringer J (2006). Reverse Genetics. WormBook.

[B56] Chalfie M, Sulston J (1981). Developmental genetics of the mechanosensory neurons of Caenorhabditis elegans. Dev Biol.

[B57] Bateman A, Coin L, Durbin R, Finn RD, Hollich V, Griffiths-Jones S, Khanna A, Marshall M, Moxon S, Sonnhammer EL (2004). The Pfam protein families database. Nucleic Acids Res.

